# Group II Metabotropic Glutamate Receptor Agonist LY379268 Regulates AMPA Receptor Trafficking in Prefrontal Cortical Neurons

**DOI:** 10.1371/journal.pone.0061787

**Published:** 2013-04-12

**Authors:** Min-Juan Wang, Yan-Chun Li, Melissa A. Snyder, Huaixing Wang, Feng Li, Wen-Jun Gao

**Affiliations:** 1 Department of Neurobiology and Anatomy, Drexel University College of Medicine, Philadelphia, Pennsylvania, United States of America; 2 Department of Neurobiology and Anatomy, Zhongshan College of Medicine, Sun Yat-Sen University, Guangzhou, China; Institut National de la Santé et de la Recherche Médicale (INSERM U901), France

## Abstract

Group II metabotropic glutamate receptor (mGluR) agonists have emerged as potential treatment drugs for schizophrenia and other neurological disorders, whereas the mechanisms involved remain elusive. Here we examined the effects of LY379268 (LY37) on the expression and trafficking of the α-amino-3-hydroxy-5-methyl-4-isoxazole propionate (AMPA) receptor subunits GluA1 and GluA2 in prefrontal neurons. We show that LY37 significantly increased the surface and total expression of both GluA1 and GluA2 subunits in cultured prefrontal neurons and in vivo. This effect was mimicked by the selective mGluR2 agonist LY395756 and was blocked by mGluR2/3 antagonist LY341495. Moreover, we found that both GluA1 and GluA2 subunits were colocalized with PSD95 but not synapsin I, suggesting a postsynaptic localization. Consistently, treatment with LY37 significantly increased the amplitude, but not frequency, of miniature excitatory postsynaptic currents. Further, actinomycin-D blocked LY37's effects, suggesting a transcriptional regulation. In addition, application of glycogen synthase kinase-3beta (GSK-3β) inhibitor completely blocked LY37's effect on GluA2 surface expression, whereas GSK-3β inhibitor itself induced decreases in the surface and total protein levels of GluA1, but not GluA2 subunits. This suggests that GSK-3β differentially mediates GluA1 and GluA2 trafficking. Further, LY37 significantly increased the phosphorylation, but not total protein, of extracellular signal-regulated kinase 1/2 (ERK1/2). Neither ERK1/2 inhibitor PD98059 alone nor PD98059 combined with LY37 treatment induced changes in GluA1 or GluA2 surface expression or total protein levels. Our data thus suggest that mGluR2/3 agonist regulates postsynaptic AMPA receptors by affecting the synaptic trafficking of both GluA1 and GluA2 subunits and that the regulation is likely through ERK1/2 signaling in GluA1 and/or both ERK1/2 and GSK-3β signaling pathways in the GluA2 subunit.

## Introduction

Glutamate is the major excitatory neurotransmitter in the mammalian central nervous system. It acts through ionotropic receptors and metabotropic receptors (mGluRs). Extensive pharmacology studies have indicated that the group II mGluR2/3 receptor is associated not only with normal brain functions [1] but also with several neurological and psychiatric disorders [2]. Drugs acting on mGluR2/3 offer therapeutic benefits to patients with these disorders, especially for treatment of schizophrenia [3]–[7]. Clinical trial studies reported that treatment with the mGluR2/3 agonist LY2140023 led to significant improvements in both positive and negative symptoms in patients with schizophrenia, and its analogue LY379268 (LY37) reversed the behavioral effects in animal models of schizophrenia [7]. mGluR2/3 agonists also reversed some physiological and behavioral dysfunctions induced by *N*-methyl-d-aspartate (NMDA) receptor antagonists in animal models [8]–[13] and human subjects [14]. Our recent study suggested that LY37 can reverse the disrupted NMDA receptor expression induced by the NMDA receptor antagonist dizocilpine (MK-801) [15].

The therapeutic potential of mGluR2/3 agonists in schizophrenia is certainly intriguing, but it is unclear whether an mGluR2/3 receptor agonist also has regulatory effects on AMPA receptor expression. AMPA receptors mediate most of the excitatory synaptic transmission in the brain and underlying synaptic plasticity and behavior [16], [17]. The glutamate hypothesis of schizophrenia posits decreased signaling through NMDA, and possibly AMPA, subtypes of glutamate receptors [18], [19]. The mRNA expression of AMPA receptor subunits was altered [19]–[22] and AMPA receptor subunits GluA1-4, especially the GluA2 subunit, were reduced [23], [24] in the brains of patients with schizophrenia. Recent evidence also suggests abnormal trafficking of AMPA receptors in this illness [23], [25]. We therefore hypothesize that mGluR2/3 agonist, used as a non-dopaminergic antipsychotic drug for treatment of schizophrenia [3], [26], might directly affect the expression and/or trafficking of AMPA receptors in the postsynaptic site. To test this possibility, we treated cultured prefrontal cortex (PFC) neurons with the mGluR2/3 agonist LY37 and examined the extracellular membrane protein levels of GluA1 and GluA2 subunits, as well as the mechanisms involved in their regulation. We found that LY37 significantly increased GluA1 and GluA2 surface expression and this effect involved transcription and was differentially regulated via ERK1/2 and GSK-3β signaling pathways.

## Materials and Methods

### Animal treatment and primary PFC neuron culture

Animal procedures were in accordance with the National Institutes of Health (NIH) animal use guidelines. The experimental protocols were approved by the Institutional Animal Care and Use Committee at Drexel University College of Medicine.

Timed pregnant Sprague-Dawley rats were purchased from Charles River Laboratories (Wilmington, MA). The primary PFC neuron cultures were prepared from embryonic day 18 (E18) rats. PFC tissues were removed from embryos in cold dissection medium [10 mM HEPES, 33.3 mM glucose, 12 mM MgSO_4_, 5 µg/ml gentamicin, and 0.3% bovine serum albumin (BSA) in Hank's buffered salt solution (HBSS)] and dissociated with trypsin (Gibco, Carlsbad, CA) at 37°C, filtered by cell filter (BD Biosciences, San Jose, CA). Neurons were rinsed with neurobasal media (which contains 0.5% glutamine, 100 µg/ml penicillin, and 100 µg/ml streptomycin) supplemented with B27 and 5% fetal bovine serum (FBS, Gibco). Neurons were then plated in growth medium (neurobasal media plus 2% B27 supplement and 5% FBS) on 6-well plates coated with poly-d-lysine (5 µg/cm2, BD Biosciences, Franklin Lakes, NJ) at a density of 100,000 cells/cm2 at 37°C with 5% CO2 for 4 h. Cells were then maintained in neurobasal medium supplemented with B27 and l-glutamine without FBS at 37°C /5% CO_2_. Cells were fed 2 times per week, with half of the media changed each time.

### Immunofluorescence Staining and Image Analysis

Neurons at 17–18 days in vitro (DIV) were treated with LY37 (Tocris Bioscience, Minneapolis, MN) at different concentrations (0.1, 1, 10, and 100 µM) or with culture medium as vehicle control for 1 h. To label the surface expression, we used N-terminal antibodies against GluA1 or GluA2 subunits, respectively. The labeling was performed as previously described [27]–[29]. Briefly, PFC neurons were washed with cold 0.1 M phosphate-buffered saline (PBS) and fixed with 4% paraformaldehyde/4% sucrose in 0.1M PBS for 10 min at room temperature. Neurons were then blocked with 10% BSA in 0.1 M PBS for 1 h at room temperature and incubated in the following dilutions of primary antibodies overnight at 4°C: monoclonal rabbit anti-GluA1 N-terminal and anti-GluA2 N-terminal (1∶100, Millipore, Billerica, MA). After rinsing with 0.1 M PBS for several times, cells were incubated in Dylight 488 or 594 secondary (1∶800, Jackson ImmunoResearch Laboratories, West Grove, PA) for 1 h at room temperature under nonpermeable conditions. After washing with 0.1 M PBS, coverslips were mounted in 50% glycerol containing a high quality anti-fade medium Mowiol® 4–88 (Polysciences, Inc., Warrington, PA) and DABCO (1,4-diazabicyclo (2,2,2)octane, Sigma-Aldrich, St. Louis, MO).

To examine the colocalization of GluA1 or GluA2 with postsynaptic density (PSD95) or Synapsin I, surface GluA1 or GluA2 was labeled similar to the procedure described above. Cells were incubated with Dylight -594 or Alexa-488 conjugated secondary antibody (1∶800, Jackson ImmunoResearch), followed by permeabilization with 0.3% Triton X-100 for 10 min at room temperature. The cells were then incubated with either anti- PSD95 or anti-Synapsin I goat-anti rabbit monoclonal antibody overnight at 4°C (1∶500, Millipore, Billerica, MA). Cells were incubated with goat-anti rabbit secondary antibody (Alexa-488 for PSD95 or Dylight-594 for Synapsin I) to visualize puncta.

Images were acquired with a 60x/1.4 NA oil Apochromatic objective using a Zeiss Axiovert 200M inverted microscope with AxioCam MR camera via Axiovision software (Carl Zeiss, Inc. North America, Thornwood, NY). All groups were analyzed simultaneously using cells from the same culture preparation. For each experimental group, more than 20 cells were selected from at least three different wells, and approximately 5 to 8 cells from each well were analyzed. Dendritic segments (20 µm/segment, 2–3 segments/neuron) located about one soma diameter away from the soma were selected and the average of the dendritic segments was used to represent each neuron. Single surface labeling quantification was performed using ImageJ software (Rasband, W.S., ImageJ, U. S. National Institutes of Health, Bethesda, MD, http://imagej.nih.gov/ij/, 1997–2012)[30]. The colocalization analysis was measured with the plugin “Intensity correlation analysis” of ImageJ software. Quantification of colocalization was done automatically and consistently by this software. In every experiment, the red fluorescence was detected in channel 1 (Ch1), and the green fluorescence was primarily detected in channel 2 (Ch2). In all calculations, intensities were subtracted with background. The threshold of each channel as well as the background was determined by a default algorithm set (pixel = 3) in the software to remove any bias attributable to visual interpretation and random errors. Pixels below the threshold were eliminated for the purpose of quantifying colocalization. Region of interest (ROI) was chosen in channel 1. Image layers selected for analysis were converted into 8-bit monochromatic images. Fluorescence intensity was quantified in matched ROIs for each pair of images. Pearson's correlation coefficient (Rr values shown in the Result) was calculated and analyzed for colocalization [31], [32]. Statistical analysis was performed using one-way analysis of variance (ANOVA) for multiple-comparisons among the several experimental groups and then with Student t-test between the controls and individual drug treatment group. All data were presented as mean±standard error. Images were prepared for printing with Adobe Photoshop (San Jose, CA) and Canvas (ACD Systems, Ltd., Victoria, BC, Canada).

### BS^3^ Cross-linking Assay and Western Blotting

Bis (sulfosuccinimidyl) suberate (BS^3^) cross-linking was performed as described previously [33]–[35]. BS^3^ is a membrane-impermeable agent, which selectively cross-links cell surface proteins to form high-molecular-mass aggregates. Because intracellular proteins are not modified, they retain normal molecular mass. This enables surface and intracellular pools of a particular protein to be distinguished by sodium dodecyl sulfate–polyacrylamide gel electrophoresis (SDS–PAGE) and Western blotting. To detect surface and intracellular protein, culture neurons at DIV17–18 were treated with LY37 (1 µM), or ERK1/2 inhibitor PD98059 (50 µM, Abcam, Cambridge, MA) for 1 h prior to treatment with LY37 (1 µM). The media were then removed and the plates were rinsed 3 times with 0.1 M PBS. BS^3^ cross-linker was then added to the plates containing HBSS for 30 min at room temperature. The BS^3^ cross-linker was diluted in PBS (20 mM sodium phosphate, 0.15 M NaCl, pH 8.0) with a final concentration of 1 mM. Quench solution (20 mM Tris, pH 7.5) was then added to a final concentration of 20 mM and incubated for 15 min at room temperature. Neurons were scrapped in radioimmunoprecipitation assay (RIPA) buffer, which contains 50 mM Tris, 20 mM Tris-HCl (pH 7.5), 150 mM NaCl, 1 mM EDTA,1 mM EGTA, 1% sodium deoxycholate, 2.5 mM sodium pyrophosphate, 20 mM beta-glycerophosphate disodium salt hydrate, 1 mM Na_3_VO_4_, 1 µg/ml leupeptin, 0.1% SDS, 1% Triton, 1 mM phenylmethanesulfonylfluoride (PMSF), and 1 mM NaF. The collected neurons were homogenized and the homogenate was centrifuged at 10,000×*g* for 10 min at 4°C. The supernatant was transferred to a new microcentrifuge tube and kept at −20°C.

### In vivo treatment with LY37

To test whether LY37 also affects GluA1 or GluA2 expression in vivo, animals (male SD rats at postnatal day 90) were administered with LY279268 (0.3 mg/kg, intraperitoneal injection) or saline vehicle (control, n = 6 each group) and were sacrificed after 60 min. Brain tissue containing mPFC were quickly removed and sectioned as 400 µm slices with a Vibratome, and were incubated with BS^3^ (1 mg/ml; Pierce Biotechnology, Rockford, IL, USA) in aerated artificial cerebrospinal fluid (ACSF, 95% O_2_ and 5% CO_2_) at 4°C for 40 minutes with gentle agitation. The slices were then washed three times with ice-cold ACSF containing 20 mM Tris (pH 7.6) to quench the remaining BS^3^ and the surface protein of GluA1 and GluA2 was determined by Western blot analysis.

The protein concentration was measured using a protein assay kit (Bio-Rad Laboratories, Hercules, CA). Samples were boiled at 95°C with sample buffer containing β-mercaptoethanol. Protein samples were subjected to 7.5% SDS-PAGE and then transferred to 0.45 µm polyvinylidene fluoride transfer membranes (Millipore). Membranes were blocked with 5% nonfat dry milk in TBST (0.05% Tween-20 in 1×Tris-buffered saline) for 1 h and were incubated in the following dilutions of primary antibodies overnight at 4°C: monoclonal mouse anti-GluA1 (1∶2000, Millipore), rabbit anti-GluA2 (1∶2000, Millipore), monoclonal mouse anti-actin (1∶20,000, Sigma-Aldrich), and rabbit anti-p44/42 MAPK (Erk1/2), anti-p44/42 MAPK(Erk1/2), and Thr202/Tyr204 (1∶2000–4000, Cell Signaling Technology, Danvers, MA). After several rinses with TBST, the membranes were incubated in horseradish peroxidase–conjugated goat anti-mouse or rabbit IgG (Jackson ImmunoResearch Laboratories) at 1∶6000 for 1 h at room temperature. The immunopositive protein bands were detected with ECL Western Blotting System (GE Healthcare, Pittsburgh, PA) and exposed to HyBlot CL autoradiography film (Denville Scientific Inc., Metuchen, NJ). Blots were scanned and band densities were quantified with ImageJ software. Total proteins were directly normalized to the levels of β-actin which was set as 100%, whereas phosphorylation proteins were first normalized to corresponding total proteins and then to the control levels. Samples from each group were obtained from 3 to 4 independent experiments, and neurons in 3 wells (in 6-well plates) from each group were collected together as an experimental sample. Each sample was analyzed at least 4 times to reduce interblot variability. Thus 3 to 4 independent experiments were done for each test, yielding 12 to 16 blot bands for data analysis. The results are presented as mean±standard error. Significance was determined with the Student *t* test with 95% confidence. Images were prepared for printing with Adobe Photoshop and Canvas.

### Patch Clamp Recordings in Neuronal Cultures

Similarly, primary neuron cultures were prepared from embryonic day 18 rat PFC. Cultured PFC neurons at DIV 15-18 were treated with either culture medium as control or LY37 (1 µM) for 1 h at 37°C, and then used for recording of AMPA receptor-mediated mEPSCs at ∼35°C under voltage clamp mode. The external perfusion solution contained (in mM) 140 NaCl, 5 KCl,, 2 CaCl2, 10 glucose,10 HEPES, 1 MgSO_4_, 0.0005 tetrodotoxin, pH 7.4. The membrane potential of the recorded neurons was held at −60 mV during recording in the presence of NMDA receptor antagonist APV (50 µM, Tocris Bioscience, Minneapolis, MN) and GABA_A_ antagonist picrotoxin (100 µM, Tocris Bioscience, Minneapolis, MN) to block NMDA and GABA_A_ receptors, respectively. A Cs^+^ internal solution was used for recording and it contained the following ingredients (in mM) 120 Cs-gluconate, 5 lidocaine (QX-314), 6 CsCl2, 1 ATP-Mg, 0.2 Na2GTP, and 10 HEPES at pH 7.3 (adjusted with CsOH). The mEPSCs recorded in voltage-clamp mode were analyzed with Clampfit 9.2 (Molecular Devices). After recording, a typical mEPSC was selected to create a sample template for event detection within a data period. The frequency (event number) and amplitude of individual events were examined with Clampfit (Molecular Devices, Sunnyvale, CA). The data were analyzed by Student's t test and were presented as mean±SE.

## Results

### Group II mGluR agonist LY37 increases surface expression of GluA1 and GluA2 subunits in cultured prefrontal neurons

To examine the effects of LY37, a selective mGlu2/3 receptor agonist, on AMPA receptors' surface expression in PFC neurons, we used immunochemical staining of 17–18 DIV cultured neurons with antibodies directed against the extracellular N-terminal of GluA1 or GluA2 subtypes. Live neurons were treated with LY37 at dosages from 0.1 to 100 µM or with control medium for 1 h at 37°C. Surface GluA1 or GluA2 receptors were labeled with N-terminal antibodies under nonpermeable conditions [27], [28].

**Figure 1:**
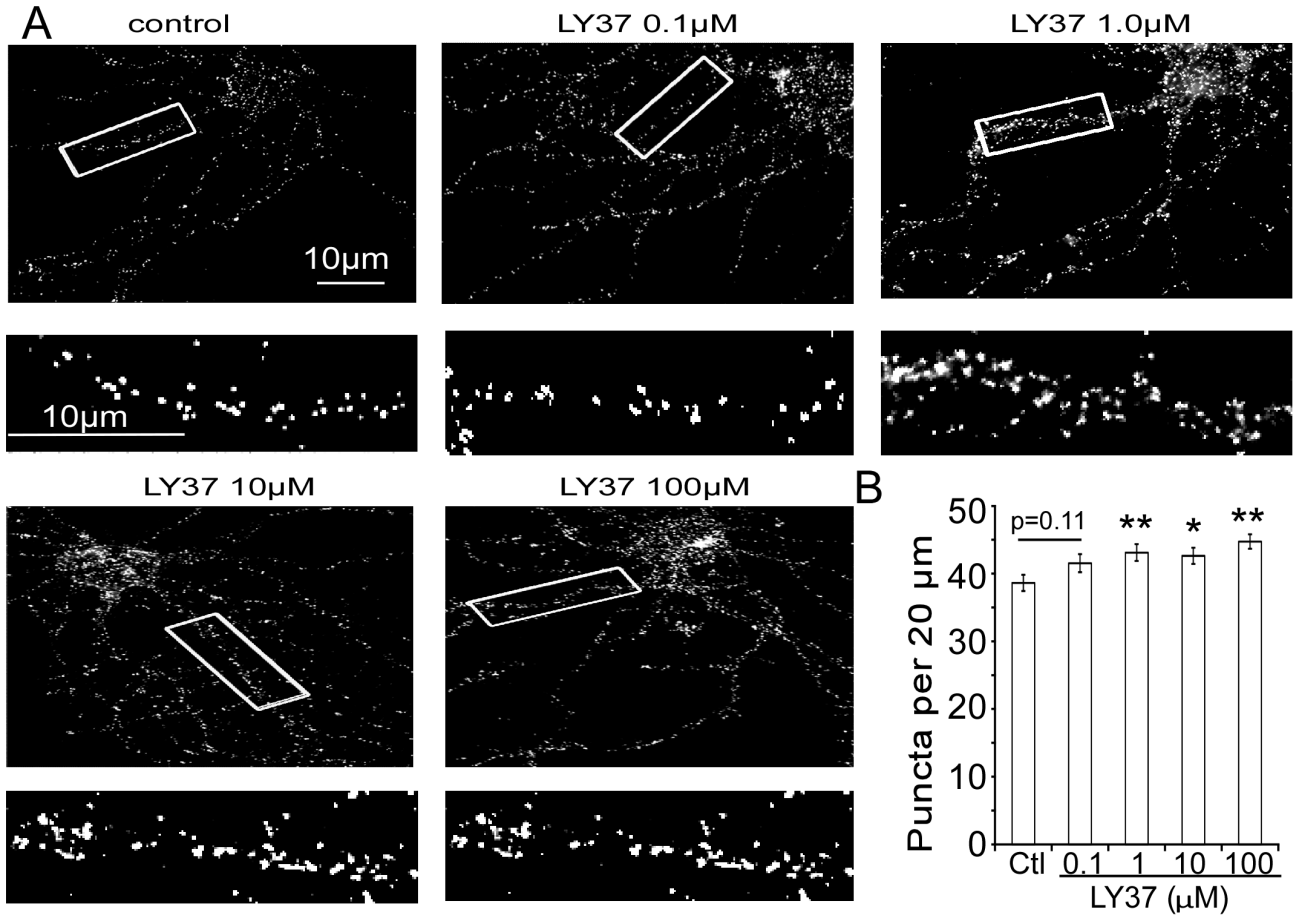
The mGluR2/3 agonist LY37 induces a significant increase in surface expression of GluA1 subunits in cultured PFC neurons. (A) Representative immunofluorescent images of GluA1 receptor surface expression in response to a 60 min exposure to 0.1, 1, 10 or 100 µM LY37 treatment. LY37 at doses of 1, 10 and 100 µM increased the surface expression, i.e., cluster density, of GluA1 subunits on the dendrites. Images were taken by microscope at 63×oil lens. Scale bar = 10 µm for upper panels and for the enlarged images in the lower panels. (B) Pooled data show that surface puncta density of GluA1 was significantly increased by LY37 at doses of 1.0 to 100 µM (n = 20 neurons for control and for each doses, * p<0.05, ** p<0.01; ANOVA F = 3.501, p<0.01).

As shown in Figure 1, we found that LY37 induces a modest (7.8% to 16.9%) but consistent increase in surface expression of GluA1 subunits in cultured PFC neurons. Representative immunofluorescent staining images of GluA1 receptor surface expression in response to different doses of LY37 treatment are shown in Figure 1A. LY37 treatment (1 h) increased the surface expression density, i.e., cluster numbers per 20 µm, of GluA1 subunits on the neuronal dendrites. Pooled data demonstrate that LY37 treatments increased the surface puncta numbers of GluA1 subunits at all dosages from 0.1 to 100 µM, but the cluster numbers were significantly increased only at the higher doses of 1.0 to 100 µM compared with vehicle controls (control∶ 38.6±1.2; 0.1 µM LY37: 41.6±1.3; 1 µM LY37∶ 43.1±1.2; 10 µM LY37∶ 42.6±1.2; 100 µM LY37∶ 44.7 ±1.1; n = 20 neurons for control and for each dose, * p<0.05, ** p<0.01; ANOVA F = 3.501, p<0.01; Fig. 1B).

**Figure 2:**
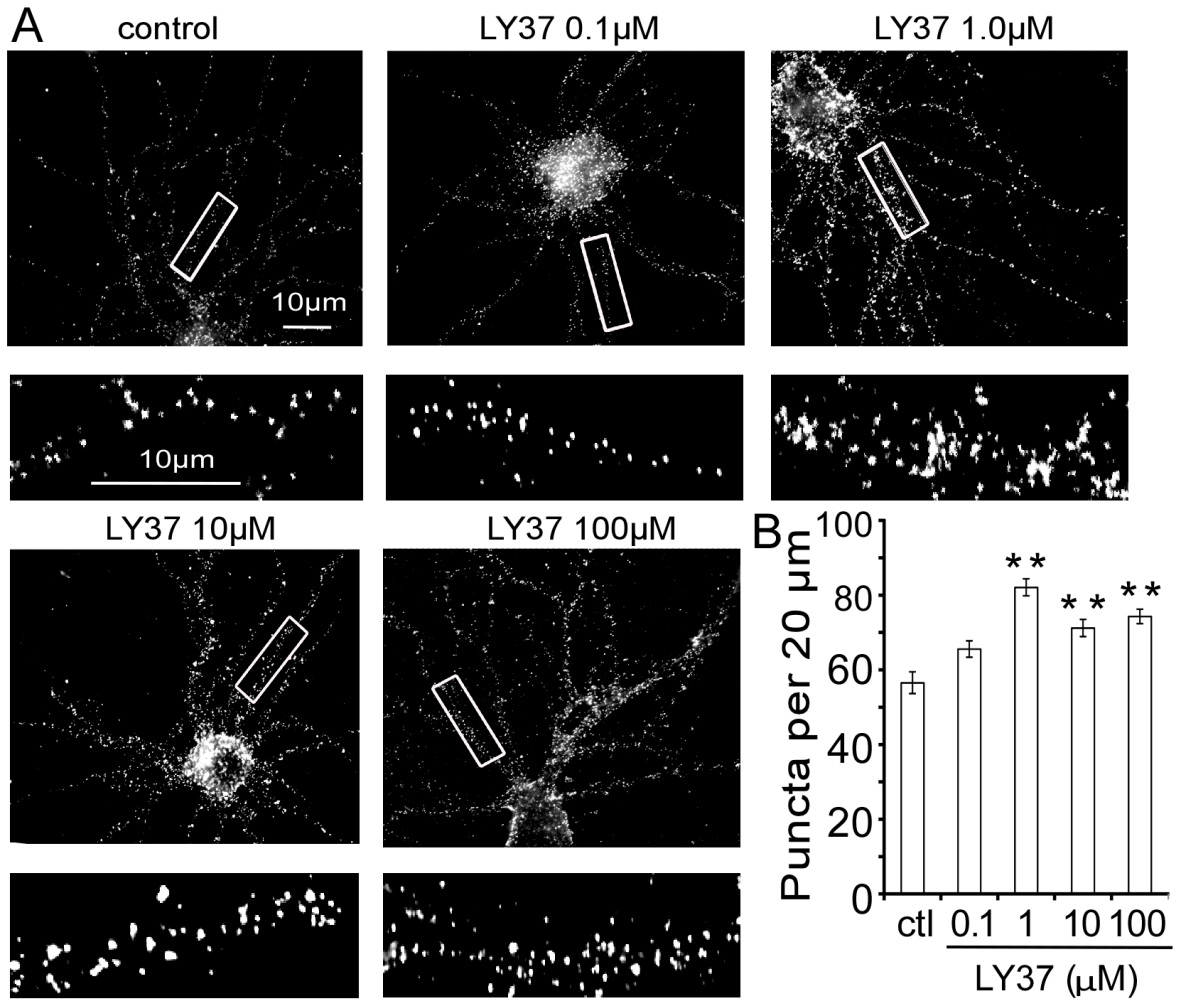
LY37 increased GluA2 surface expression in the cultured PFC neurons. (A) Representative immunofluorescent images of GluA2 receptor surface expression in response to a 60 min exposure to 0.1, 1, 10, or 100 µM LY37 treatment. Culture medium served as a vehicle control. Scale bar = 10 µm for both upper panels and for the enlarged images in the lower panels. (B) Bar graphs show that GluA2 receptor surface puncta density on the neuronal dendrites was significantly increased by LY37 at concentrations from 1 to 100 µM (n = 20 neurons for each group, ** p<0.01). However, at lower doses of 0.01 and 0.1 µM, LY37 did not induce clear alterations in cluster numbers in GluA2 subunits (p > 0.05; ANOVA F = 5.288, p<0.001).

Similarly, LY37 increased GluA2 surface puncta density in cultured PFC neurons. As shown in Figure 2, PFC neurons at 17–18 DIV were treated with LY37 of 0.1, 1, 10, or 100 µM for 1 h at 37°C with culture medium as vehicle control (Figure 2A). Summary bar graphs show that GluA2 receptor surface puncta number on the neuronal dendrites was significantly increased 26.0% to 45.3% by LY37 at concentrations from 1 to 100 µM (control∶ 56.5±2.9; 1 µM LY37∶ 82.1±2.3; 10 µM LY37∶ 71.2±1.9; 100 µM LY37∶ 74.3±2.7; n = 20 neurons for each group, ** p<0.01; ANOVA F = 5.288, p<0.001; Fig. 2B). However, at lower dose of 0.1 µM, LY37 did not induce clear alterations in cluster numbers of GluA2 subunits on the dendrites of PFC neurons (0.1 µM LY37∶ 65.6±2.3; p > 0.05; Fig. 2B).

### Treatment with LY37 increased protein levels of both total and surface GluA1 and GluA2 subunits in cultured prefrontal neurons and these effects were mimicked by selective mGluR2 agonist LY395756

**Figure 3:**
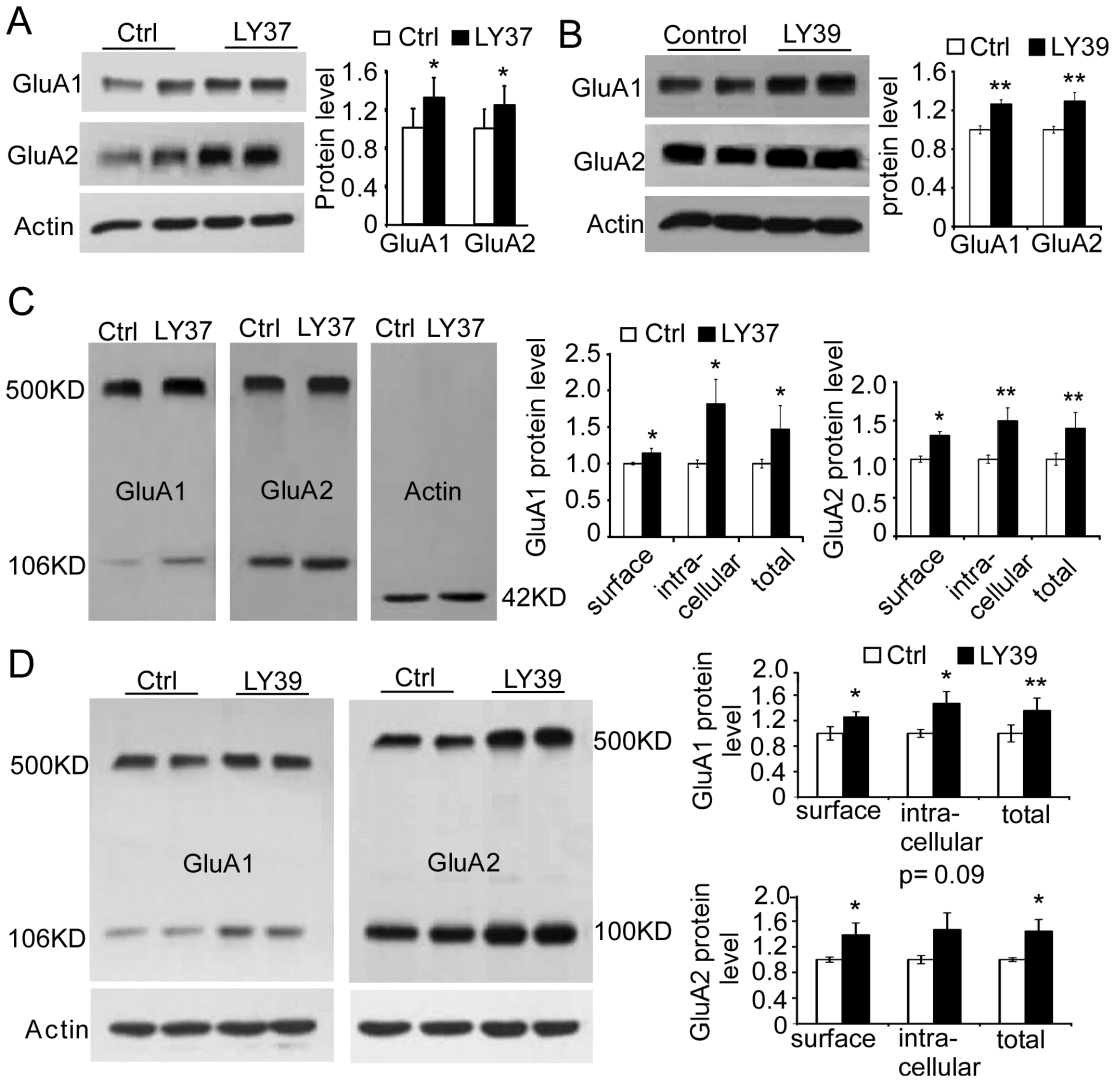
LY37 and selective mGluR2 agonist LY395756 (LY39) increased both surface and total protein level of GluA1 and GluA2 subunits in the cultured prefrontal neurons. (A and B) Treatment with LY37 or LY395756 significantly increased the total protein levels of both GluA1 and GluA2 subunits (*p<0.05, **p<0.01, 6 independent experiments). (C) Representative immunoblots (left) show the surface and intracellular components of GluA1 and GluA2 subunits that were separated with BS^3^ cross-linker (right and middle panel). The surface proteins cross-linked with BS^3^ and formed large molecules at ∼500 KD, which were easily separated from the small intracellular components (∼100 KD) by Western Blot. In contrast, actin was detected only in 42KD because it is highly enriched in the cytosol but not in the cell membrane. Summary bar graphs on the right show the relative changes of surface and intracellular components of GluA1 and GluA2 subunits, respectively. LY37 (1 µM) treatment significantly increased the surface expression of both GluA1 and GluA2 (*p<0.05, 4 independent experiments). The intracellular and total protein levels of GluA2 subunit were also significantly increased by LY37 (*p<0.05, ** p<0.01). However, neither intracellular nor total protein level of GluA1 exhibited significance although there was a trend of increases (p > 0.05). (D) Presence of LY39 (1 µM) for 1 h in 17–18 DIV PFC neurons in incubator elevated the surface, intracellular, and total protein levels of GluA1 significantly; whereas the surface and total protein levels, but not the intracellular protein level, of GluA2 were significantly increased (*p<0.05, **p<0.01, 4 independent experiments).

Because treatment with LY37 at 1 µM and higher dosages significantly increased the surface puncta density of both GluA1 and GluA2 subunits in the cultured prefrontal neurons (17–18 DIV), we wondered whether activation of mGLuR2/3 would not only affect surface but also total protein levels of GluA1 and GluA2 subunits. We first examined total protein levels of GluA1 and GluA2 using Western blotting. Cultured PFC neurons (17–18 DIV) were treated with LY37 (1 µM) for 1 h and were then collected in lysis buffer for protein assay. As shown in Figure 3A, the total protein level of both GluA1 and GluA2 subunits was significantly increased by treatment with LY37 (*p<0.05, Fig. 3A).

LY37 is a highly selective group II mGluR agonist with EC_50_ values at 2.69 and 4.48 nM for mGluR2 and mGluR3, respectively (Tocris Bioscience). Recent studies report that the mGluR2 subunit, but not the mGluR3 subunit, is responsible for the actions of the mGluR2/3 agonist [12], [36]–[38]. We therefore tested the effects of the mGluR2 agonist (1*S*,2*S*,4*R*,5*R*,6*S*)-rel-2-Amino-4-methylbicyclo[3.1.0]hexane-2,6-dicarboxylic acid (LY395756) on GluA1 and GluA2 expression. LY395756 is a selective metabotropic glutamate ligand for mGluR2 and mGluR3 receptors with K_i_ values of 0.165 and 0.302 µM, respectively (Tocris Bioscience). It acts oppositely, however, as an agonist at mGluR2 (EC50 = 0.397±0.039 µM) and antagonist at mGluR3 (IC50 = 2.94±0.20 µM) [39]. We therefore used a concentration of 1 µM, as a recent study reported [37], to treat cultured prefrontal neurons (17–18 DIV) at 37°C for 1 h. The neurons were then lysed for a protein assay and Western blot, as in the experiments conducted for mGluR2/3 agonist LY37. As shown in Figure 3B, the total protein levels of both GluA1 and GluA2 were significantly increased by application of LY395756 (**p<0.01, Fig. 3B).

Next we used a membrane-impermeable protein cross-linker BS^3^ to separate surface and intracellular receptors. The surface proteins cross-linked with BS^3^ and formed large molecular congregate at ∼500 kD, which were easily separated from the small intracellular components (∼100 kD) when detected with Western blot. This technique allowed us to determine whether the changes in surface protein level of GluA1 and GluA2 in response to mGluR2/3 agonist LY37 were derived from alterations of both intracellular and total protein levels. Similarly, cultured prefrontal neurons were initially treated with LY37 (1 µM) for 1 h and then were incubated with BS^3^ cross-linker at room temperature for 30 min after several rinses with 0.1 M PBS. As shown in Figure 3C (left), the surface proteins of both GluA1 and GluA2 were linked with BS^3^ to form a 500-kD large molecule, whereas the intracellular proteins retained normal molecular mass and were thus separated with a 106-kD and 100-kD band, respectively. β-actin is highly enriched in the cytosol, the Western blot band was located in 42 kD only without a large molecular (500-kD) band (Fig. 3C, left). This clean 42-kD band served as a control for the reliability of the technique. We found that GluA1 surface protein level was significantly increased by LY37 (*p = 0.028), as well as intracellular and total protein levels (the latter was calculated as surface plus intracellular protein). (*p = 0.035 and 0.014, respectively, Fig. 3C right). Consistently, the levels of surface, intracellular, and total protein levels of GluA2 subunits were all significantly increased by treatment with LY37 (*p<0.05, ** p<0.01; Fig. 3C right).

Next, we incubated cultured PFC neurons with BS^3^ cross-linker at room temperature for 30 min after treatment with LY395756 (1 µM) for 1 h at 37°C. Likewise, surface proteins were separated from intracellular proteins by BS^3^ and the total protein level was calculated as surface protein plus intracellular protein. We found that the surface, intracellular, and total protein levels of GluA1 were all significantly increased by LY395756 (p = 0.02, 0.02, and 0.002, respectively; Fig. 3D). Also, both surface and total protein levels of GluA2 were significantly elevated (p = 0.02 and 0.01, respectively; Fig. 3D), but the intracellular protein showed a trend of increase without statistical significance (p = 0.09; Fig. 3D).

### LY37-induced changes in GluA1 and GluA2 subunits were effectively blocked by mGluR2/3 antagonist LY341495

**Figure 4:**
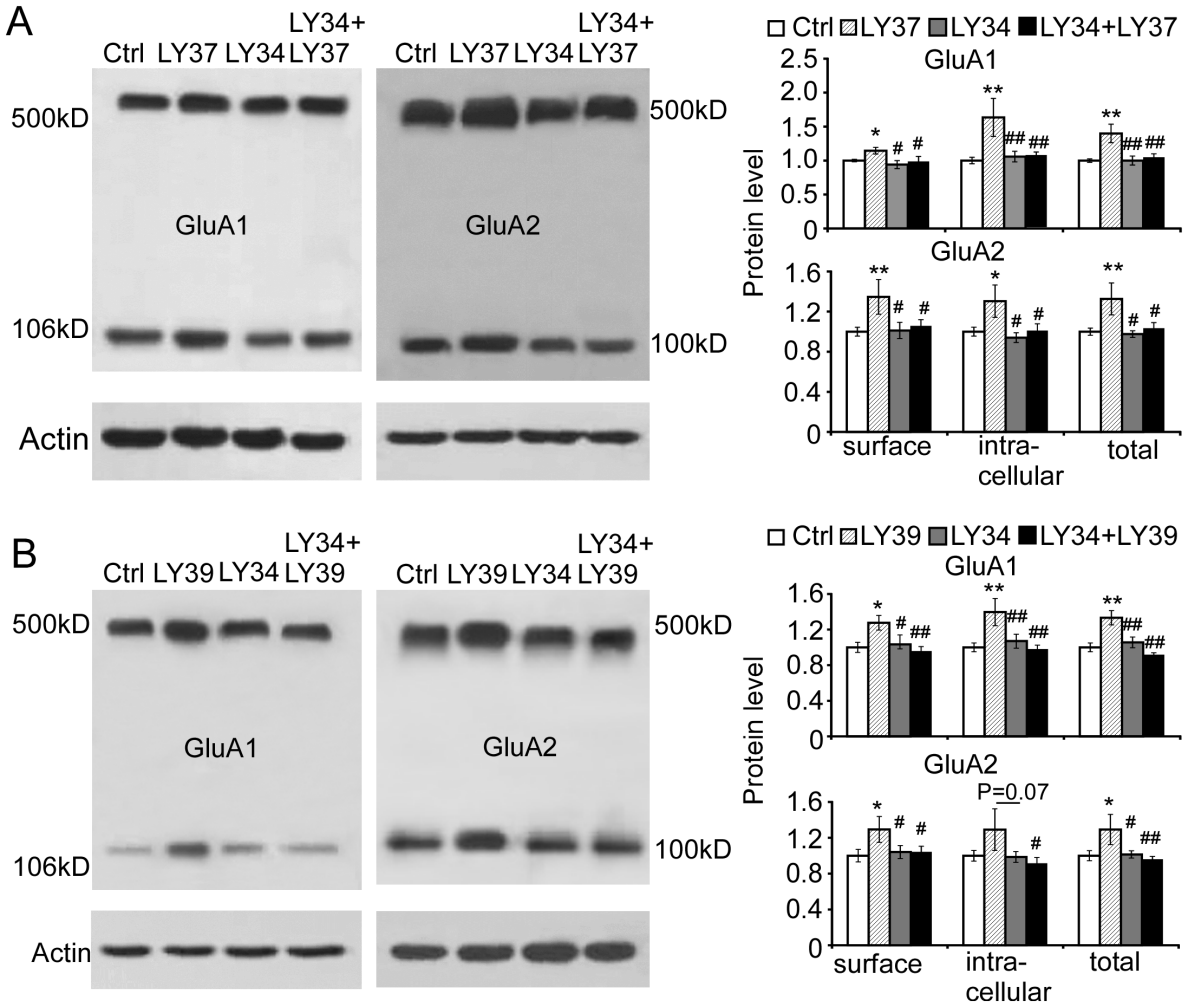
Selective mGluR2/3 antagonist LY341495 (LY34, 100 nM) completely blocked the effects of LY37 and LY395756 (LY39) on the protein levels of both GluA1 and GluA2 subunits. (A) LY37 signficantly increased the expression of GluA1 and GluA2 receptors, including surface, intracellular and total proteins (* p<0.05, ** p<0.01 compared with control). Further, whereas mGluR2/3 antagonist LY34 itself did not induce changes in protein levels of GluA1 or GluA2, LY34 completely blocked LY37's effects on protein levels of both GluA1 and GluA2 subunits when it was applied 30 min prior to LY37 treatment (p > 0.05 for all compared with control; # p<0.05, ## p<0.01 compared with LY37; 6 independent experiments). (B) Compared with control, LY39 signficantly increased the protein levels of surface, intracellular and total GluA1 and GluA2 receptors (* p<0.05, ** p<0.01) whereas LY34 itself did not induce changes in the protein levels of GluA1 and GluA2 receptors (p > 0.05 for all). However, LY34 effectively blocked LY39's effects on protein levels of both GluA1 and GluA2 (; # p<0.05, ## p<0.01 compared with LY39; 6 independent experiments).

To further confirm the action of mGluR2/3 agonist LY37, we pretreated cultured prefrontal neurons with a highly potent and selective group II antagonist (2*S*)-2-Amino-2-[(1*S*,2*S*)-2-carboxycy cloprop-1-yl]-3-(xanth-9-yl) propanoic acid (LY341495, Tocris Bioscience, Minneapolis, MN; 100 nM) 30 min before application of LY37. As shown in Figure 4A, LY37 signficantly increased the expression of both GluA1 and GluA2, including surface, intracellular, and total proteins (* p<0.05, ** p<0.01 compared with control). Further, whereas LY341495 (LY34) itself did not induce any changes in the protein levels of both GluA1 and GluA2 subunits (p > 0.05 for all, Fig. 4A), LY34 completely blocked the increase in both GluA1 and GluA2 protein levels induced by LY37 (p > 0.05 for all when compared with control; # p< 0.05, ## p<0.01 compared with LY37; Fig. 4A). Additionally, the mGluR2 agonist LY395756 (LY39, 1 µM for 1 h at 37°C) significantly increased the expression of both GluA1 and GluA2 (* p<0.05, ** p<0.01 compared with control). In contrast, pretreatment with LY341495 (1 µM) for 30 min also completely blocked the effects of LY39 on the surface and total protein levels of both GluA1 and GluA2 subunits (p > 0.05 for all), while LY341495 itself did not induce any significant change in either GluA1 or GluA2 protein levels (p > 0.05 for all compared with control; # p< 0.05, ## p<0.01 compared with LY39; Fig. 4B). These data suggest that LY37 affects GluA1 and GluA2 expression by acting through mGluR2.

**Figure 5:**
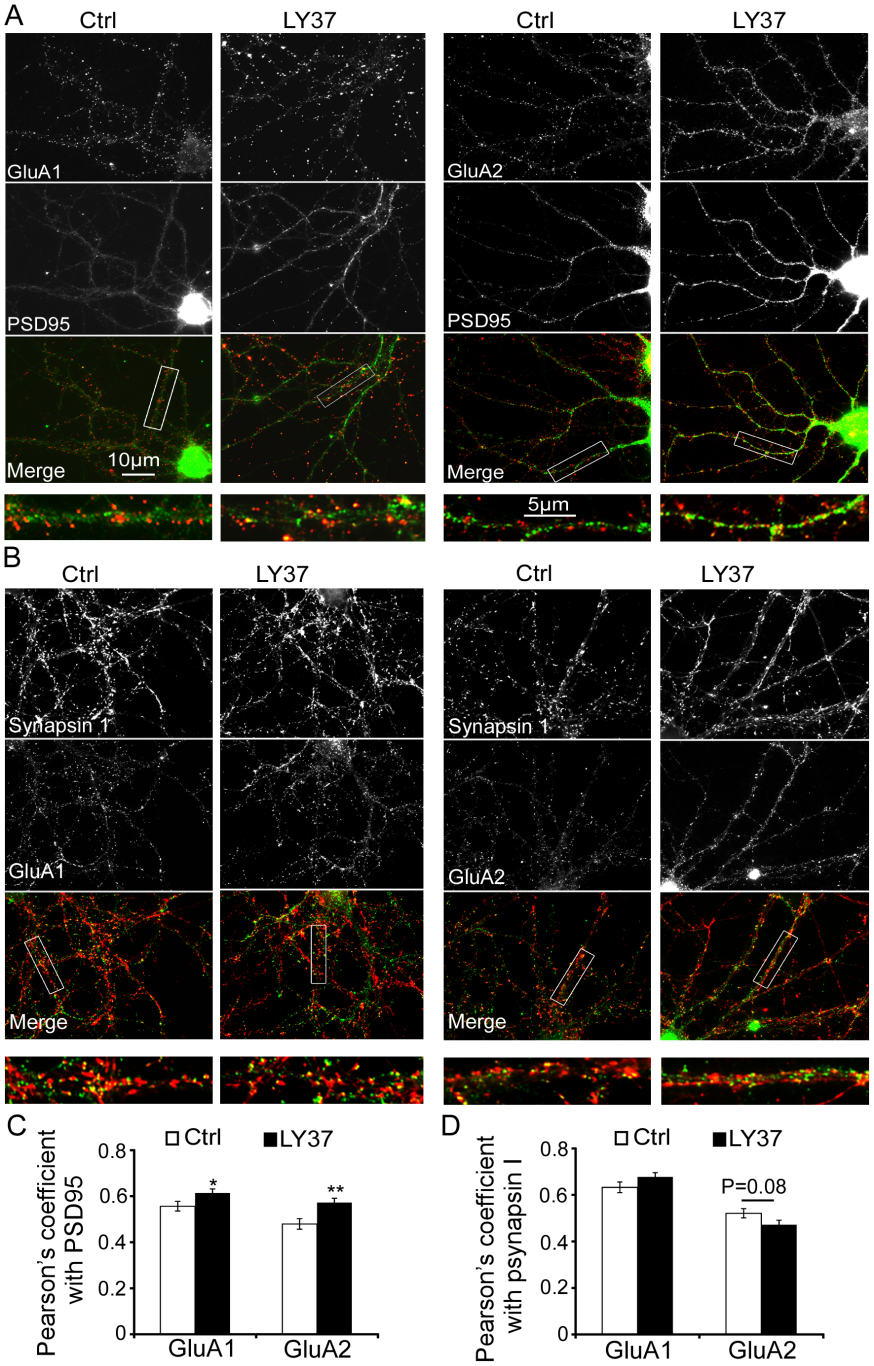
LY37 increased GluA1 or GluA2 colocalization with PSD95 but not synapsin I. (A) Immunofluorescent images show colocalization of surface GluA1 or GluA2 receptor (red: Dylight-594) with PSD95 (green: Alexa-594) in DIV15–17 primary PFC neurons. Scale bar = 10 µm for most of the images in A and B and scale bar = 5 µm in the enlarged images. (B) Coimmunolabeling of GluA1 (green: Alexa-488; control: n = 38 neurons, LY: n = 36), GluA2 (green: Alexa-488; control: n = 20, LY: n = 22) and synapsin I (red: Dylight-594) expression in cultured primary PFC neurons. (C) Bar graph shows the Pearson's correlation coefficients, in which LY37 (1 µM, 1 h) significantly increased the colocalization percentages of both GluA1 (control: n = 24, LY37: n = 31) and GluA2 surface receptors with PSD95 (control: n = 35, LY37: n = 39; *p< 0.05, **p<0.01). (D) Bar graph shows the percent colocalization of GluA1 or GluA2 with psynapsin I in neurons with or without LY37 treatment. The Pearson's correlation coefficient was similar, without significance for both GluA1 and GluA2 (p > 0.05 for both).

**Figure 6:**
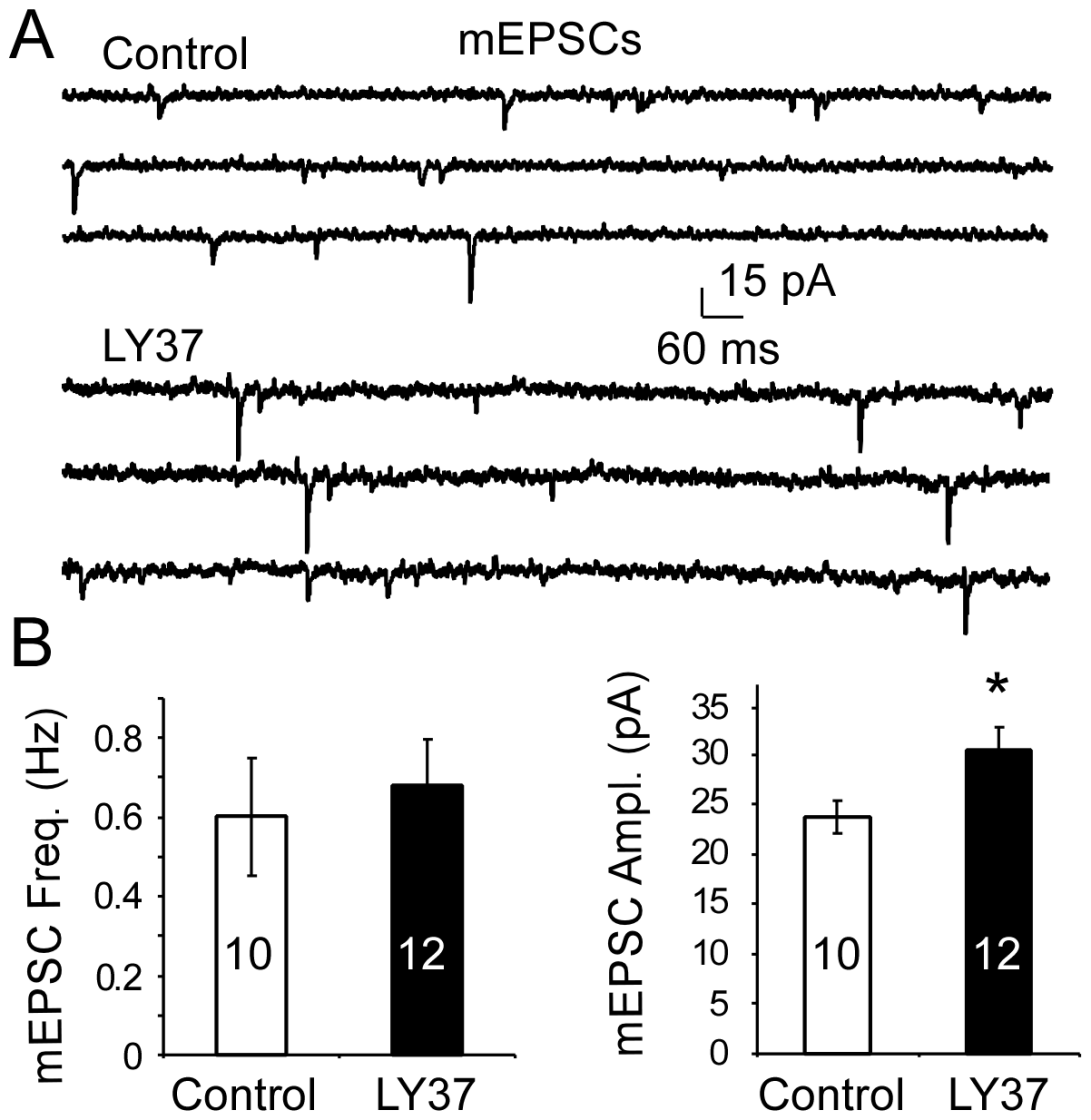
LY37 increased amplitude of AMPA receptor-mediated miniature excitatory postsynaptic currents (mEPSCs). (A) Representative traces of mEPSCs were recorded from 15–18 DIV PFC neurons with (n = 12) or without (n = 10) treatment of LY37 (B) Bar graphs show that the amplitude of mEPSCs was significantly increased (*p<0.05; left) but the frequency of mEPSCs was unchanged (p > 0.05; Right) after treatment with LY37 (1 µM) for 1 hour at 37°C with culture medium.

To further examine whether LY37-induced surface expression of GluA1 and GluA2 receptors are trafficked into synapses, we conducted two more experiments. First, we examined the colocalization of surface GluA1 or GluA2 with PSD95 or Synapsin I. as shown in Figure 5, Pearson's correlation of coefficient (R^2^) was used to measure the overlap and correlation of fluorescent intensity in two different staining. We found that the percent colocalization of GluA1 or GluA2 with PSD95 was significantly increased by LY37 treatment (1 µM, control∶ R^2^ = 0.556±0.02, n = 24; LY∶ R^2^ = 0.614±0.02, n = 31; p = 0.04 for GluA1; control∶ R^2^ = 0.480±0.02, n = 35; LY: 0.573±0.02, n = 39; p = 0.002 GluA2; Fig. 5A and C). In contrast, there was no significant difference in the colocalization between GluA1 and Synapsin I (control: R^2^ = 0.633±0.02 n =  38; LY: R^2^ = 0.677±0.03; n = 36, p = 0.2) and between GluA2 and Synapsin I (control: R^2^ = 0.521±0.02 n =  20, LY: R^2^ = 0.472±0.02; n = 22, p = 0.08, Fig. 5B and D). Second, we recorded the mEPSCs in cultured PFC neurons treated with LY378268 (1 µM, 1 h) compared with those in culture medium. We found that, as shown in Figure 5, LY378268 significantly increased the amplitude (p<0.05) but not frequency (p > 0.05) of mEPSCs (n = 10 for control and n = 12 for LY; Fig. 6A and B). These data suggest that the increased GluA1 and GluA2 receptors induced by LY37 are not only localized in synapses but also functional.

### LY37-induced increase of GluA1 and GluA2 expression may be invovled in transcription

**Figure 7:**
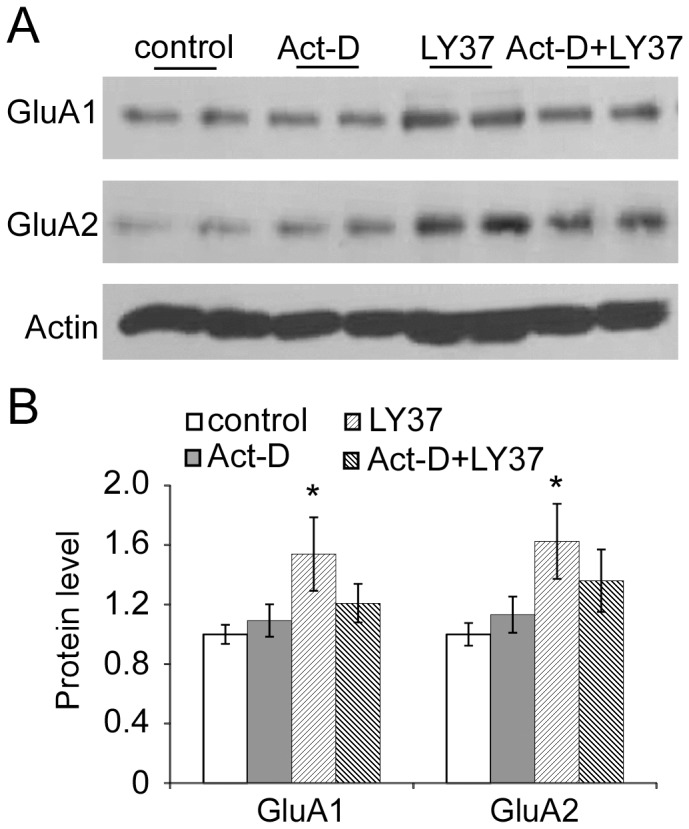
Actinomycin-D partially blocks the effects of LY37 on total protein levels of GluA1 and GluA2 receptors. (A) Representative blots of GluA1 and GluA2 in cultured PFC neurons in the presence or absence of Act-D (10 µg/ml, 4 h) prior to LY37 (1 µM, 1 h) treatment. (B) Compared with control, LY37 significantly increased total protein levels of GluA1 and GluA2 (* p<0.05 compared with control), whereas prior treatment with Actinomycin-D partially blocked these effects (*p >* 0.05 compared with control, 4 independent experiments).

Given the increases in total protein and the long time course of LY37 application (1 h), we examined whether the increases in total protein require transcription. Neurons were treated with the transcriptional inhibitor actinomycin D (Act-D, 10 µg/ml) for 4 h at 37°C, as previous studies reported [40], [41] before application of LY37 (1 µM, 1 h). As shown in Figure 7, we found that LY37 significantly increase both GluA1 and GluA2 expressions (* p<0.05 for both, 4 independent experiments) but these effects were partially blocked when neurons were treated with Act-D prior to LY37 application (p > 0.05 for both, Fig. 7). These results suggest that the LY37-induced increase in GluA1 and GluA2 expression may involve regulation of transcription although further exploration is needed.

### LY37 increased surface, intracellular and total protein levels of GluA1 and GluA2 receptors in vivo

**Figure 8:**
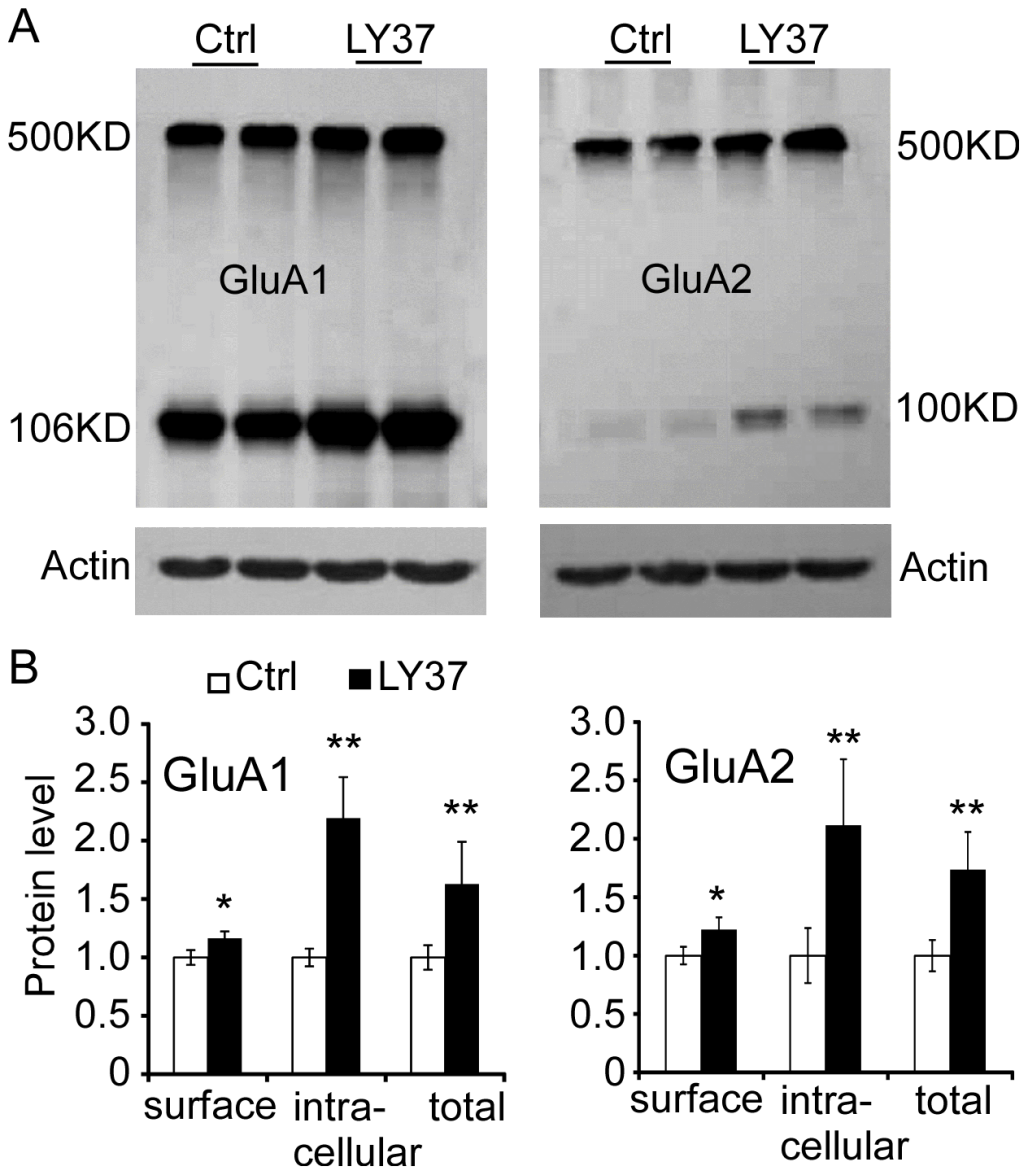
Both GluA1 and GluA2 receptors, including surface, intracellular and total protein expressions, are significantly increased after the animals (P90 male SD rats) were treated with LY37 (0.3 mg/kg, i.p. injection, 1 h). (A) Representative images show the GluA1 and GluA2 surface and intracellular receptors were separated by BS^3^ after gel electrophoresis. (B) Summary data show that LY37 in vivo injection significantly increased the expression levels in surface, intracellular, and total proteins of GluA1 and GluA2 (*p<0.05, **p<0.01; n = 6).

We wondered whether LY37 could have similar effects on GluA1 and GluA2 protein levels in vivo. Male adult SD rats were injected with LY37 (0.3 mg/kg, i.p.) and after 1 h, brains were removed, sliced into 400 µm, and incubated with BS^3^. The surface and intracellular receptors were separated by gel electrophoresis. Similarly, we found that LY37 significantly increases the surface, intracellular, and total GluA1 and GluA2 protein levels (* p<0.05, ** p<0.01; n = 6; Fig. 8).

### GSK-3β-specific inhibitor TDZD exhibited differential effects on GluA1 and GluA2 subunit changes induced by LY37 treatment in cultured PFC neurons

**Figure 9:**
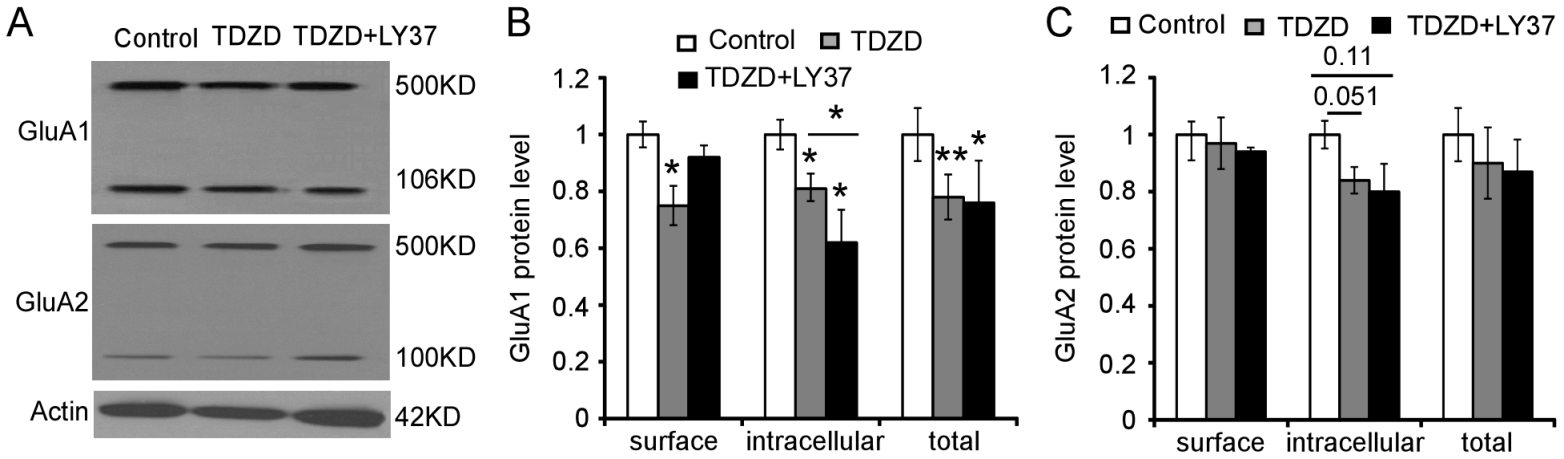
Specific GSK-3β inhibitor TDZD exhibited differential effects on the GluA1 and GluA2 subunit changes induced by LY37 treatment in cultured PFC neurons. (A) Representative Western blot images show the surface and intracellular components of GluA1 and GluA2 subunits that were separated with BS^3^ cross-linker. (B and C) Summary bar graphs show the relative changes of surface and intracellular components of GluA1 and GluA2 subunits, respectively. It appears that TDZD itself significantly decreased the surface, intracellular, and total protein levels of GluA1 subunits but had no effects on the protein levels of GluA2 subunits (*p<0.05, ** p<0.01). However, when TDZD and LY were treated together simultaneously, the GluA1 expression, including surface, intracellular, and total protein levels, recovered to the control levels without significant differences (p > 0.05 for all). There were also no changes in GluA2 protein level when TDZD and LY were applied together (p > 0.05 for all). These data indicate that GSK-3β may not mediate the trafficking of GluA1 but is involved in the expression and trafficking of GluA2 receptor subunits.

How does the mGluR2/3 agonist affect the trafficking of AMPA receptors? In our recent study, we found that mGluR2/3 agonist not only has antipsychotic efficacy similar to that of a D2 antipsychotic agent but also shares the same signaling pathway, i.e., GSK-3β signaling, in the regulation of NMDA receptor expression in the prefrontal neurons [15]. We therefore tested whether the mGluR2/3 agonist LY37 also regulates the surface expression of GluA1 and GluA2 subunits via activation of GSK-3β signaling. As shown in Figure 9, the surface and intracellular components of GluA1 and GluA2 subunits were separated with BS^3^ cross-linker. We found that the GSK-3β inhibitor thiadiazolidinones (TDZD) itself (10 µM for 1 h) significantly decreased the surface, intracellular, and total protein levels of GluA1 subunits (*p<0.05, ** p<0.01; Figure 9A and B). This is in agreement with a recent study in which application of GSK-3 inhibitors or knockdown of GSK-3 caused a significant reduction of the amplitude of miniature excitatory postsynaptic current (mEPSC), a readout of the unitary strength of synaptic AMPARs [42]. Moreover, when TDZD and LY37 were applied together, the GluA1 surface protein level, but not the total protein level, was restored to the control levels, without significant differences (p > 0.05). However, intracellular protein levels of GluA1 subunits were not reversed but decreased significantly compared with TDZD itself (*p<0.05, ** p<0.01; Fig. 9B). In contrast, TDZD itself did not induce any effects on the GluA2 subunits, including surface, intracellular, and total protein levels. Interestingly, even when TDZD and LY37 were applied together, we found no significant changes in the GluA2 subunits (Fig. 9A and C). These data indicate that GSK-3β inhibitor itself affects the trafficking of GluA1 subunits. Therefore, the effect of GSK-3β inhibitor on LY37-induced GluA1 protein level could not be determined by GSK-3β inhibitor's own effects on GluA1. However, GSK-3β inhibition completely blocked the protein synthesis and trafficking of GluA2 subunits induced by LY37 although the mechanism remains to be explored.

### The mGluR2/3 receptor agonist LY37 increases AMPA receptors' surface expression through activating ERK1/2

**Figure 10:**
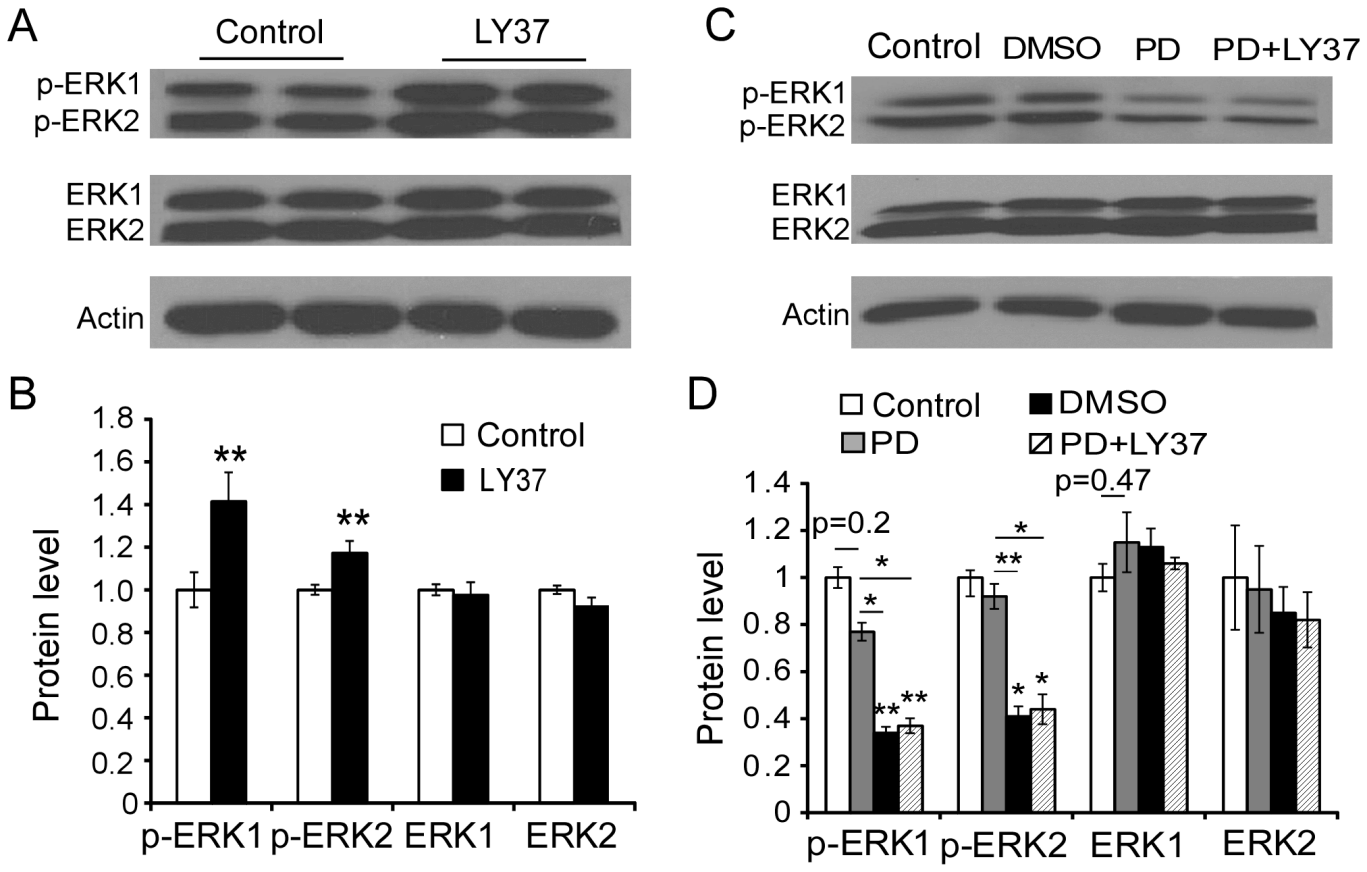
LY37 treatment significantly increased the phosphorylation of ERK1/2 activity in the cultured PFC neurons, and this was prevented by the ERK inhibitor PD98059 (PD). (A) Representative images of ERK1/2 and p-ERK1/2 expression in response to LY37, PD (50 µM for 1 h) or PD followed by LY37 treatment (1 µM for 1 h), DMSO as PD's vehicle. (B) LY37 administration did not induce clear changes in either ERK1 or ERK2 total protein level (p > 0.05). However, it significantly increased the expression of both p-ERK1 and p-ERK2 ratios (**p<0.01). PD significantly decreased both p-ERK1 and p-ERK2 protein ratios (*p <0.05, ** p <0.01) but not on the total protein levels of either ERK1 or ERK2 (P > 0.05 for both). With application of PD98059, LY37 did not affect the protein levels of p-ERK1 and p-ERK2 nor reverse the effects of PD98059.

Because the role of GSK-3β in regulation of GluA1 and GluA2 subunits is complicated by its inhibitor's direct reduction of GluA1 expression, we wondered whether LY37 regulates GluA1 and GluA2 subunits through different signaling pathways. Previous studies showed that p42/44 MAPK (ERK1/2) activation can drive synaptic delivery of AMPA receptors [43] and mGluR2/3 agonist can activate ERK1/2 [44], [45]. We speculated that mGluR2/3 agonist may instead enhance AMPA receptor, particularly GluA1, surface expression through the MAPK-ERK pathway if not via GSK-3β signaling. To test this possibility, we examined p42/44 MAPK activity by analyzing the phosphorylation (activation) level of ERK1/2 using Western blot. Primary cultured PFC neurons at 17–18 DIV were exposed to LY37 (1 µM) for 1 h at 37°C. After treatment, cells were collected, total protein levels of ERK1/2 and phosphorylation levels were then investigated. As shown in Figure 10A and B, LY37 induced significant increases in levels of both p-ERK1 (p = 0.0004) and p-ERK2 (p = 0.009). In contrast, LY37 did not have significant effects on the total protein level of either ERK1 or ERK2 (p > 0.05 for both, Fig. 10A and B). These results indicated that LY37 induced a marked ERK1/2 activation in phosphorylation but had no effect on ERK1 and ERK2 total protein levels.

To further investigate whether ERK1/2 signaling is regulated by LY37, the neurons were exposed to ERK1/2 inhibitor 2-(2-Amino-3-methoxyphenyl)-4*H*-1-be nzopyran-4-one (PD98059; 50 µM; Tocris Bioscience) for 1 h or PD98059 (50 µM) + LY37 (1 µM) for 1 h with 0.02% DMSO as vehicle and culture medium as control. We found that DMSO itself slightly decreased p-ERK1 level but this effect was not significant. We saw no clear effects on p-ERK2 level or on the total protein levels of ERK1 and ERK2. In contrast, PD98059 induced significant decreases of both p-ERK1 and p-ERK2 without effects on the total protein levels of either ERK1 or ERK2 (*p <0.05, ** p <0.01; Fig. 10C and D). However, after ERK1/2 activity was blocked by the selective inhibitor PD98059 (50 µM for 1 h), LY37 application (1 µM for 1 h) did not induce clear changes in protein levels of either p-ERK1/2 or ERK1/2 compared with the changes observed with PD98059 treatment alone (p > 0.05) or with LY37 alone (p > 0.05). These results confirmed the effectiveness of ERK1/2 inhibitor PD98059 in blocking the action of LY37 on the p-ERK1 and p-ERK2.

**Figure 11:**
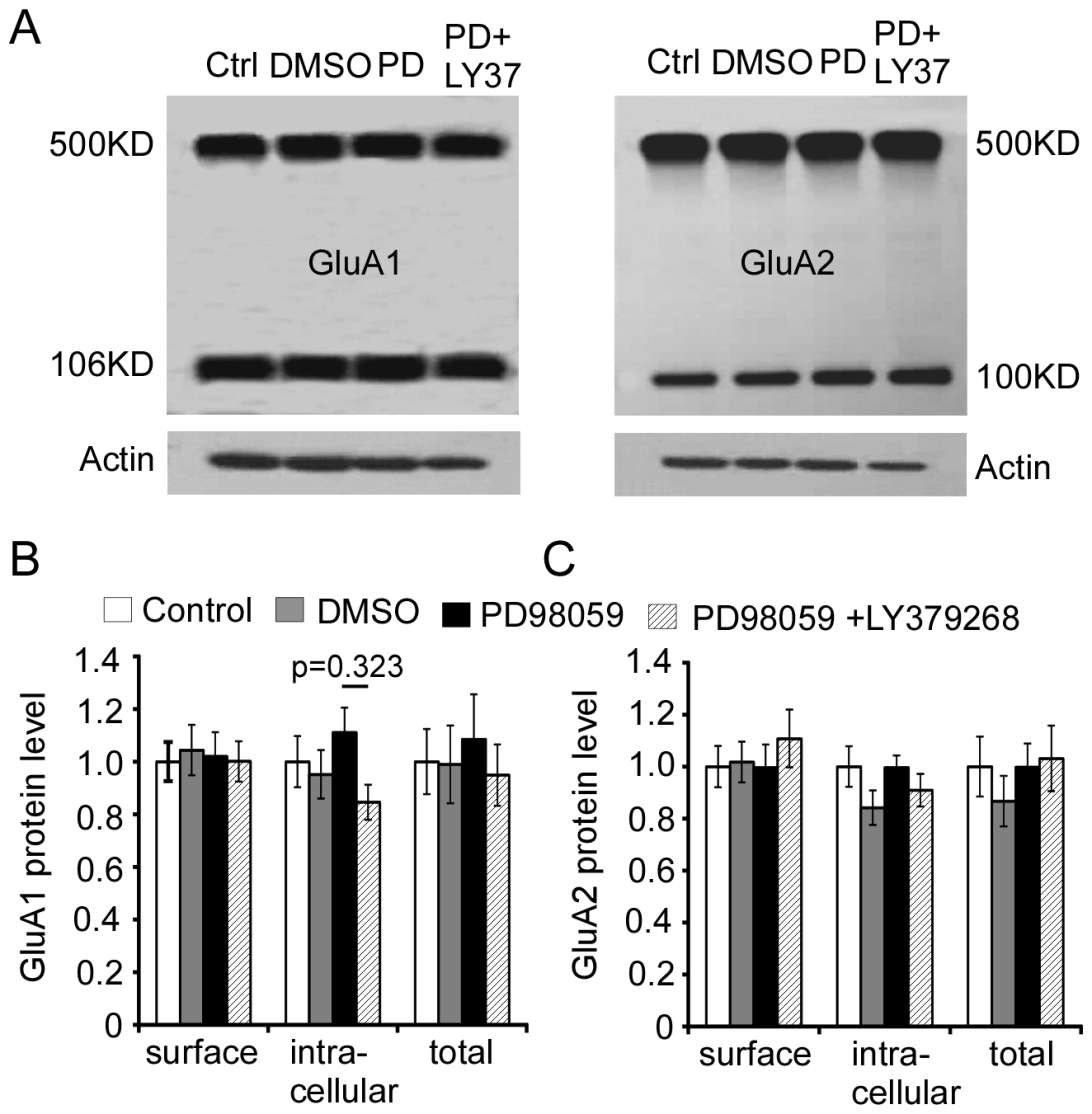
ERK1/2 inhibitor PD98059 prevents the increases in surface levels of both GluA1 and GluA2 subunits induced by LY37 treatment in cultured PFC neurons. (A) Representative images of drug treatment. Primary cultured PFC neurons at 17–18 DIV were exposed to DMSO (0.02%), LY37 (1 µM), ERK inhibitor PD98059 (50 µM), or PD98059 (50 µM) + LY37 (1 µM) for 1 h at 37°C, respectively. After treatments, the cells were incubated in BS^3^ cross-linker and the protein levels on the membrane surface and intracellular components were then separated by Western blot. (B and C) Summary bar graphs show the protein levels of surface and intracellular GluA1 and GluA2 subunits in different treatments groups compared to vehicle controls. Neither ERK1/2 inhibitor PD98059 nor PD98059+LY37 treatment induced changes in GluA1 and GluA2 surface expression (p > 0.05 for all comparisons).

Moreover, we confirmed that mGluR2/3 agonist LY37-induced trafficking of AMPA receptors is indeed mediated through ERK1/2 activation. We tested whether activation of p-ERK1/2 is required for the delivery of AMPA receptors from intracellular to extracellular membranes in response to LY37 treatment. Primary cultured PFC neurons at 17–18 DIV were exposed to DMSO (0.02% as vehicle control), PD98059 (50 µM) alone, or PD98059 (50 µM) 1 h prior to LY37 (1 µM) for 1 h at 37°C, respectively. After treatment, cells were incubated in BS^3^ cross-linker for 30 min at room temperature. The loaded protein samples were tested with N-terminal antibodies against GluA1 and GluA2, respectively, to examine the surface protein level. The protein level on the membrane surface and intracellular components was then separated by Western blot. We found that there were no significant changes in either GluA1 or GluA2 surface level (p > 0.05 for both, Fig. 11) after these treatments. Summary bar graphs (Fig. 11B) show the protein levels of surface and intracellular GluA1 and GluA2 subunits in different treatments groups compared with DMSO and control. Neither ERK1/2 inhibitor PD98059, nor PD98059+LY37 treatment, induced changes in GluA1 and GluA2 surface, intracellular, or total protein levels under these conditions (p > 0.05 for all comparisons). These data indicate that the ERK1/2 inhibitor PD98059 prevents the increases in surface expression of both GluA1 and GluA2 subunits induced by LY37 treatment in cultured PFC neurons.

## Discussion

In this study, we provided evidence that an mGluR2/3 agonist, and also a specific mGluR2 agonist, directly regulate postsynaptic AMPA receptors by affecting the synaptic trafficking of both GluA1 and GluA2 subunits in cultured prefrontal cortical neurons and in vivo. In addition, we also showed that the effects induced by the mGluR2/3 agonist were mediated by activation of ERK1/2 for GluA1 trafficking, and both ERK1/2 and GSK-3β signaling pathways for GluA2 trafficking.

In the last decade, both group I and II mGluRs have emerged as potential therapeutic targets for various neurological and neuropsychiatric disorders [2], [46]–[49], including schizophrenia [3], [5], [50]–[53], anxiety and stress disorder [47], [54], [55], and depression [56]. In particular, the mGluR2/3 agonist appears to be a novel therapeutic target with great potential for treatment of schizophrenia.

Schizophrenia is a devastating psychiatric illness that afflicts about 1% of the world population. The core symptoms in schizophrenic patients include positive symptoms (thought disorder, delusions, hallucinations, paranoia), negative symptoms (social withdrawal, anhedonia, apathy, paucity of speech), and cognitive deficits (impairments in perception, attention, learning, short- and long-term memory, and executive function) [57]. The cognitive deficits in schizophrenia are one of the major disabilities associated with the illness and are considered a reliable predictor of long-term disability and treatment outcome [58]–[62]. While currently available antipsychotics effectively treat the positive symptoms, they offer only limited effects on the negative symptoms and cognitive impairments. Furthermore, some patients are unresponsive to current antipsychotic treatments and some agents exhibit major adverse side effects, including disturbances in motor function, weight gain, and sexual dysfunction [58], [59]. Thus, there is a substantial need to develop more effective therapeutics for this debilitating disorder that provide major improvements in efficacy across multiple symptoms and have fewer adverse effects.

A major challenge in developing novel therapeutic approaches for treatment of schizophrenia is the absence of clear molecular or cellular pathological changes responsible for this disorder. However, the last two decades have led to an increased awareness of the importance of the negative symptoms and cognitive impairments in patients with schizophrenia and their resistance to dopamine D2 receptor antagonism. These insights have led to a reformulation of the classical dopamine hypothesis [57], [62]–[64] and to formulation of an abnormal glutamate hypothesis in the pathophysiology of schizophrenia [2], [18], [47], [65]. This progress presents exciting opportunities for rational discovery of therapeutics for schizophrenia.

Preclinical trial studies indicated that mGluR2/3 agonists exhibited great efficiency in alleviating both positive and negative symptoms of schizophrenia with limited adverse side effects [7], [12], [66]–[68] although a recent Phase 2 trial exhibited a discouraging finding [69]. Activation of mGluR2/3 also reverses phencyclidine (PCP)/MK-801-induced disruption of neuronal activity [10], decreases neuronal injury produced by MK-801 [70], and reduces ketamine-evoked glutamate, but not dopamine, release in the PFC in animal models [71], [72]. Importantly, mGluR2/3 agonists also seem to be effective in preventing certain aspects of cognitive impairment [14], [51], [73]–[77] although studies of mGluR2/3 agonists in animal models have not produced concrete evidence of cognitive benefit [67], [78].

Despite compelling behavioral data and results from a recent preclinical trial [7], the cellular mechanisms by which activation of mGluR2/3 attenuates the effects of NMDAR antagonism and neurological disorders remain elusive. Many researchers speculate that mGluR2/3 agonists may attenuate symptoms by reducing excessive glutamate release through presynaptic mechanisms in key areas of the brain associated with fear and anxiety, such as the PFC, amygdala, hippocampus, and thalamus [7], [52], [59], [79], [80]. However, the mGluR2/3 receptors are localized not only in presynaptic terminals but also in postsynaptic sites and glia in the cerebral cortex and limbic regions [52], [81]–[85]. Indeed, several studies reported a group II mGluR agonist-induced LTD through a possible intracellular signaling pathway in the postsynaptic cell [86]–[88]. We recently also reported that mGluR2/3 agonist significantly reverses the dysfunctional NMDA receptor expression in the MK-801 model of schizophrenia, indicating a direct postsynaptic action [15].

In this study, we investigated the surface and total protein levels of both GluA1 and GluA2 subunits in response to mGluR2/3 agonist LY37 and selective mGluR2 agonist LY395756 in the cultured prefrontal neurons. We found that the surface cluster puncta density of both GluA1 and GluA2 subunits on the cell membrane were significantly increased by application of LY37 in a dose-dependent manner. Both surface and total protein levels were significantly increased by treatment with LY37 and these effects were completely blocked by the mGluR2/3 antagonist LY341495. Moreover, recent studies indicated that mGluR2, but not the mGlu3 receptor subtype, mediates the actions of the mGluR2/3 agonist in the PCP model of schizophrenia [12], [36], [38]. We therefore tested the action of LY395756, a selective mGluR2 agonist [37], on the protein level of GluA1 and GluA2 in cultured PFC neurons. Interestingly, LY395756 exhibited effects almost identical to those of LY37 on GluA1 and GluA2 expression and trafficking. Our data thus support previous reports [12], [36], [38], indicating that the effect of LY37 on AMPA receptor subtype GluA1 and GluA2 expression and trafficking is mainly dependent on the functional mGluR2 subtype. However, our data differ from those studies that suggesting an apparent LTD induction by activation of group II mGluR agonist in pyramidal neurons of rat prefrontal cortical slices [86]–[88]. The reason for this discrepancy is not clear but it may be derived from different preparation or agonist concentration.

The question is whether GluA1 and GluA2 subunits are trafficked into synapses. Our data indicate that the puncta clusters of both GluA1 and GluA2 subunits are significantly correlated with PSD95 but not synapsin I. These data suggest that the increased GluA1 and GluA2 receptors are likely localized at synapses. This assumption is supported by the significant increase of amplitude, but not frequency, of mEPSCs in the neurons treated with LY379269. Another question is how does mGluR2/3 agonist regulate the protein expression and trafficking of AMPA receptor subunits? Given the increases in total protein and the long time course of treatment with LY37 (1 hr), we determined whether the increases in total protein require transcription. Our data indicated that transcriptional inhibitor Actinomycin D partially but significantly blocked the effects of LY37 on both GluA1 and GluA2 expression. This partially blocking effect might be attributable to the dosage and treatment time of Actinomycin D. Previous studies have reported that the inhibitory effect of Actinomycin D on transcription is dose- and time-dependent [40], [41]. It is possible that an optimal dose and treatment time with Actinomycin D may completely block the effects of LY37 on AMPA receptor trafficking but, again, further study is needed.

In addition to transcriptional regulation, posttranslational modulation also plays critical roles in glutamate receptor trafficking [89], [90]. It has been shown that the activity-dependent exocytosis of GluA1 required the ERK pathway and that diffusive ERK signaling serves as an important means by which signaling from synapses to dendritic shafts recruits AMPA receptors into synapses during LTP induction [91]. We recently also reported that an mGluR2/3 agonist can directly regulate the expression of NMDA receptors through activation of GSK-3β in the MK-801 model of schizophrenia [15]. We therefore examined the involvement of both GSK-3β and ERK1/2 pathways in LY37-induced AMPA receptor trafficking. Our data suggest that activation of GSK-3β mediated only the trafficking of GluA2 but probably not that of GluA1 because GSK-3β inhibitor itself also downregulates the expression of GluA1, in agreement with a previous study [42]. Moreover, LY37 significantly increased phosphorylation of ERK1/2 but not the total protein level of ERK1/2, indicating that ERK1/2 is also directly regulated by posttranslational modification of phosphorylation, consistent with recent studies [44], [45], [92]. Furthermore, application of the ERK1/2 upstream MEK inhibitor PD98059, which is very effective in reducing p-ERK1 and p-ERK2 [93], blocked the increase of phosphorylation of ERK1/2 and completely blocked the LY37-induced increases in surface protein levels of GluA1 and GluA2. Therefore, we believe that, in agreement with previous study of GluA2/3 [93], ERK1/2 inhibitor PD98059's effects on p-ERK might have occluded LY37's action on GluA1 and GluA2 expression. Our results thus suggest that MAPK-ERK pathway activation is required for AMPA receptors exocytosis and/or synaptic insertion. Although the exact mechanisms need further study, our data suggest that ERK1/2 but not GSK-3β is involved in GluA1 surface expression whereas both ERK1/2 and GSK-3β mediate the trafficking of GluA2 subunits.

It is generally accepted that postsynaptic AMPA receptors can be altered by synaptic activity through endocytosis and exocytosis of the receptors and plasma membranes associated with synaptic plasticity underlying the induction and maintenance of LTP [16], [94]–[96]. Synaptic plasticity induced by AMPA receptor trafficking appears to be crucial for cognitive functions such as learning and memory [17]. In terms of functional correlates of learning and memory, mGluR2 knockout mice show normal regulation of basal synaptic transmission and LTP in the hippocampus, but these animals are severely impaired in LTD induction [97]. mGluR2 activation has also been postulated to result in cognitive impairment, suggesting a potential role for mGluR2 agonist in cognitive disorders [98]. In particular, mGluR2 has been shown to selectively mediate the beneficial effects of group II agonists in rodent models of schizophrenia [3], [12], [38]. Our study indicated that mGluR2/3 agonist significantly increases the surface expression and synaptic trafficking of both GluA1 and GluA2 subunits and increases the synaptic transmission in vitro and in vivo. Whether these effects improve cognitive function and/or affect brain behaviors, and consequently achieve clinical efficacy for neuropsychiatric disorders such as schizophrenia and other diseases, remains to be determined. Nevertheless, our study provides novel evidence that mGluR2/3 agonist, in addition to its well-recognized presynaptic action, directly regulates and enhances the synaptic trafficking of AMPA receptors in the postsynaptic sites.
